# Ten important roles for academic leaders in data science

**DOI:** 10.1186/s13040-020-00228-5

**Published:** 2020-10-26

**Authors:** Jason H. Moore

**Affiliations:** Institute for Biomedical Informatics, Perelman School of Medicine, University of Pennsylvania, Philadelphia, PA 19104-6116 USA

## Abstract

Data science has emerged as an important discipline in the era of big data and biological and biomedical data mining. As such, we have seen a rapid increase in the number of data science departments, research centers, and schools. We review here ten important leadership roles for a successful academic data science chair, director, or dean. These roles include the visionary, executive, cheerleader, manager, enforcer, subordinate, educator, entrepreneur, mentor, and communicator. Examples specific to leadership in data science are given for each role.

Data science has emerged as an important discipline in the era of big data and biological and biomedical data mining. As such, we have seen a rapid increase in the number of data science departments, research centers, and schools. We review here ten important leadership roles for a successful academic data science chair, director, or dean.

## The visionary

There is no question that successful data science leaders must have a compelling vision for the future. They must have a good understanding of data science and some ideas about where the field is going over the next 5 to 10 years. Part of developing an exciting vision is knowing where the field is now and being able to extrapolate from current trends. This might be enough to develop a solid five-year vision. However, it is difficult to see 10 years into the future for a field developing and changing so rapidly. This requires synthesis and imagination. It requires asking questions about what could happen and making judgements about the probability of occurrence. Where will high-performance and cloud computing be in 10 years? What will programming languages look like in 10 years? Will artificial intelligence still be hot in 10 years? If so, what will it look like? Will we have solved all the data cleaning and integration issues we struggle with so much today? Where are the fields of statistics and applied mathematics going? Will software finally be user-friendly? What will be the important basic science, clinical, and public health questions? How much will data continue to grow in volume and complexity? What will data science education and training programs need to look like in 10 years? What can we learn from the past and how can we leverage that knowledge to enrich a vision for the future? Establishing a five- and ten-year vision is important because it becomes the basis for a plan of action required of a leader with a mandate to build and advance data science at an academic institution.

## The executive

Once a vision and a plan of action have been formulated it is then important to be able to execute that plan. This is the role of an executive. Executing a plan is largely about making decisions. Starting or growing a data science unit means hiring diverse and talented faculty and staff. Have they received the right training? Do they bring diverse viewpoints? Do they possess the skills and experience which will help the leader execute their vision? This is particularly challenging in data science because it is a new field that, to some degree, is still being defined. Further, data science means different things to different people. Because it is new it is evolving, and this presents certain strategic challenges for building a department, center, or school. For example, a leader might decide to hire faculty and staff with expertise in deep learning. What if deep learning is replaced with something else in several years? Will the unit no longer be cutting-edge? Are the faculty willing to shift their research focus to something new? These are very difficult decisions and are partly why the vision is so important. Similar challenges exist for making decisions and infrastructure and technology investments to support data science.

## The cheerleader

Executing an exciting data science vision is an important part of the job. However, an important component of successful execution is getting others excited about your vision. Important stakeholders include the funders of your effort. They must be excited about the potential impact of the data science unit on the broader institution. The faculty, staff, and students of the unit must also be excited about the vision and the plan of action because they are the ones doing the hard work required to have an impact. This means that the leader must be a cheerleader who is able to motivate hard work and innovation. Of course, all cheerleading activities must be genuine and thus trusted.

## The manager

One of the more challenging aspects of being a data science leader is the manager role. Once you have hired a diverse and talented team you must manage them. This requires assessing performance and corrective disciplinary action if necessary. It often requires managing research, teaching, and administrative space. It requires managing equitable salaries and raises tied to performance. It requires dealing with personal issues and a diversity of personalities and work preferences. A unique issue in academia is competing with the data science salaries offered by industry. This can sometimes be compensated by the advantages of working in a university environment. Advantages can include access to subsidized coursework and degree programs in addition to the rich intellectual environment with students, seminars, libraries, etc. This is important for data science because few scientists will have well-rounded training in applied mathematics, biological and biomedical sciences, computer science, informatics, and statistics. The ability to take courses and fill in expertise gaps can be appealing. The post pandemic landscape means more people will need to or want to work from home which presents managerial issues related to monitoring work hours, performance, and communication. The good news for data science is that much of the work of a data scientist can be done remotely if the employee has access to appropriate computing infrastructure locally and remotely. A successful leader should be aware of the unique challenges that come with managing data scientists.

## The enforcer

One of the most unpleasant aspects of any leadership position is enforcing university rules and regulations. This often means telling people what they can and cannot do and taking corrective or disciplinary action when the rules are broken. There are numerous rules which are specific to data science. For example, data security is a serious issue for those working with sensitive data such as those derived from electronic health records or from financial records where personal identifying information might be present. A data science leader needs to be familiar with these issues and the rules specific to their institution. These can be complex and confusing and can changes as federal laws or institutional policies change. We are also in an era where ransomware attacks are quite common. Understanding how to mitigate risks due to hacking or other types of security breaches is an important responsibility of any data science leadership position.

## The subordinate

All department chairs, center directors, and deans answer to a higher authority. Chairs and directors often report to deans and deans often report to university provosts or presidents. Sometimes interaction with a school or university board may also be necessary. It is important for a data science leader to be an able advocate for resources, hiring permissions, and structural changes with their reporting official. This often means communicating the specific data science needs to someone who does to not have a clear understanding of the discipline or its importance and impact. These relationships are very important to the success of a data science unit.

## The educator

Most academic efforts involve educating or training the next generation. One of the interesting challenges of data science education is the competition which can arise from competing units across a university. This stems from the understandable problem that everyone feels like they own or should control data science. Should data science be taught in a computer science department? Should it be taught in a department of statistics? How about informatics? Should every school (e.g. arts and sciences, business, engineering, and medicine) have their own data science unit and education program? A good data science leader needs to be able to navigate these waters during a time when everyone feels like data science stems more from their own discipline than others. There is then the debate of what data science is and what the best strategies for training data scientists are. A good leader must be flexible and understand this is a rapidly evolving discipline and there is likely no one right answer to questions regarding curriculum and practical experience.

## The entrepreneur

A leader with a vision and a plan will need resources to execute that plan. This can include money for hiring faculty and staff and money for the computational infrastructure needed by the unit or the broader university. University budgets are never as big as they need to be which means a successful data science leader needs to be involved in raising money from donors. There are several components to this role. The first is to identify specific activities or items a donor might get excited about. The second is to work with the university to assign someone in the fundraising office who can identify specific donors. The third is to be able to pitch the idea to a donor so that they can see how their generosity will benefit the university, their specific interest, or society more broadly. This is a skillset which is difficult to achieve without doing it and even seasoned data science leaders can benefit from training in the art of fundraising.

## The mentor

A good leader will inspire the next generation of data scientists and, importantly, make the time to work with them to give advice and to pass on the knowledge which comes from years of academic success and experience. Mentorship comes in many forms. For example, it could be helping a faculty member write their first data science grant. What is the best way to write the different sections of the grant? What are the most important considerations for the funding opportunity? Which review panel is best suited to review the proposal? Which federal agency has the right resources and interest in the idea? What is the best way to navigate the politics which come with any funding agency? The answers to some of these questions can be specific to data science and a good leader needs to know this. For example, a data science grant submitted to the United States (U.S.) National Library of Medicine should be more focused on advancing the discipline of data science while a data science grant submitted to a disease-specific institute of the U.S. National Institute of Health needs more of a focus on health issues. Other mentorship areas include career advice, help with networking and matchmaking, help with promotion and tenure, development of leadership skills, and assistance with securing leadership positions. Mentorship takes time but is essential to promoting the success of developing data scientists.

## The communicator

The final role is that of an effective communicator. Data science communication comes in several forms. First, as mentioned above, a leader should communicate the data science vision and plan regularly to all stakeholders. This is important so that everyone is working toward the same objectives. Second, it is important to communicate challenges and failures with as much transparency as possible without unnecessary negativity. Transparency is important because it builds trust. Trust is important when asking faculty and staff to complete tasks which are good for the unit but maybe not in the individual’s best interest. Communicating failure is important so that everyone can learn from mistakes. It is also important to move on and not dwell on mistakes. Third, it is important to be an effective communicator to the outside world. The leader is responsible to helping to build a positive data science reputation nationally or globally. This means communicating accomplishments via social media or other outlets. It could also mean communicating a positive culture and work environment. These communication efforts can help with attracting the best faculty, staff, and students as well as creating opportunities to collaborate or for fundraising. A good leader goes to the top data science conferences and networks with other leaders to discuss opportunities and challenges. Leaders love to exchange ideas and best practices and bring these back to their own institutions.

## Data Availability

Not applicable

